# Hematopoietic Cell Transplantation in Children with Sickle Cell Disease: A Single-Center Experience and Exploration of an Age Threshold for Post-Transplant Complications

**DOI:** 10.3390/jcm15208155

**Published:** 2026-10-20

**Authors:** Charlotte Nazon, Chloé Gerber, Alexandra Salmon, Annie Kamdem, Claire Pluchart, Bruno Filhon, Anne-Cécile Galoisy, Françoise Bernaudin, Catherine Paillard

**Affiliations:** 1 Department of Pediatric Onco-Hematology, Hôpitaux Universitaires de Strasbourg, 67200 Strasbourg, France; 2 Referral Center for SCD, Pediatrics Department, Intercommunal Hospital Center, 94000 Créteil, France; 3 Department of Pediatric Onco-Hematology, CHU Reims, 51092 Reims, France; 4 Department of Pediatric Hematology and Oncology, Rouen University Hospital, 76000 Rouen, France; 5 Hematology Clinical Laboratory, Hôpitaux Universitaires de Strasbourg, 67200 Strasbourg, France; 6 Laboratoire d’ImmunoRhumatologie Moléculaire, INSERM UMR_S 1109, LabEx Transplantex, Fédération de Médecine Translationnelle de Strasbourg, 67085 Strasbourg Cedex, France

**Keywords:** sickle cell disease, hematopoietic cell transplant, pediatrics

## Abstract

**Background/Objectives**: Hematopoietic cell transplantation (HCT) is the only established curative option for sickle cell disease (SCD), yet the optimal timing in pediatric cohorts remains incompletely defined. **Methods**: We retrospectively analyzed all patients under 20 years with SCD who underwent HCT at the University Hospital of Strasbourg between 2006 and 2024 and explored whether an age threshold exists for post-HCT complications. **Results**: Regarding the MSD-HCT (*n* = 30), median age at HCT was 8.4 years (3.7–16.7). All received myeloablative conditioning with thymoglobulin. Median follow-up was 4.1 years. Five-year overall and event-free survival were 93%. The single death was accidental; therefore, there was no transplant-related mortality. Grade II-IV acute GVHD occurred in two patients (7%) and chronic GVHD in one (3%). GVHD-free rejection-free survival was 87%. No graft failure occurred, and 70% achieved complete donor chimerism. No statistically significant age threshold for post-transplant complications was identified, although PRES tended to be more frequent from 8 years. Cerebral vasculopathy was stable or improved in all evaluated patients. In the haploidentical group, one primary graft failure occurred despite thiotepa and post-transplant cyclophosphamide. **Conclusions**: This cohort demonstrates excellent survival and low morbidity following MSD-HCT in children with SCD and justifies a larger registry-based analysis of age and complications. The growing role of haploidentical HCT warrants evaluation in larger prospective cohorts, with particular attention to conditioning strategies.

## 1. Introduction

Sickle cell disease (SCD) is the most common genetic disease in France. Therapeutic options, including chronic transfusion programs and hydroxycarbamide, are available to limit disease-related complications. Hematopoietic cell transplantation (HCT) remains the only established curative option; however, it is accessible to only a minority of patients. Approximately 700 allogeneic transplants were performed in France between 1988 and 2022—three quarters involving patients under 15 years of age—in a country where the SCD population is estimated at approximately 30,000 [1]. Five-year event-free survival (EFS) following matched sibling donor (MSD)-HCT using myeloablative conditioning was approximately 98% in a French series of 234 SCD patients under 30 years of age [2]. Unfortunately, fewer than 30% of SCD patients have an MSD, and the likelihood of identifying an HLA-matched unrelated donor is low, as sub-Saharan African populations are underrepresented in international bone marrow donor registries [3]. Unrelated donor HCT has been associated with unacceptable rates of graft-versus-host disease (GVHD), with a 62% rate of chronic GVHD at 1 year, including 38% extensive disease, and substantial transplant-related mortality (TRM) [4]. As a result, haploidentical donor HCT strategies using post-transplant cyclophosphamide (PT-Cy) have been developed but have shown initially an excessively high graft rejection rate of more than 40% [5]. The incorporation of thiotepa into the conditioning regimen has yielded excellent results in adults (2-yr-EFS: 94.7%) but only 68.4% in children because of a high rejection rate of 25% [6]. Moderate to severe cGVHD risk was 10%. However, in a recent French series [7], among the 13 pediatric patients, only one rejection occurred, although cGVHD and infection rates remain too high. Promising outcomes have been reported in SCD patients transplanted with ex vivo TCRαβ/CD19-depleted haploidentical-HCT, with overall survival (OS) and EFS of 86% and 83%, respectively, and efficient GVHD prevention but with an unacceptable 14% TRM related to infections [8,9]. Taken together, these findings establish haploidentical HCT as an acceptable alternative for patients lacking an MSD.

In SCD, complications tend to worsen with age, suggesting that HCT should be performed early in life, before the onset of irreversible organ damage. Several studies have highlighted an age threshold of 13–15 years old beyond which GVHD risk increases significantly [2,10,11]; however, no clear age threshold beyond which other complication risk increases significantly has been established.

This study aimed to describe the outcomes of HCT in a single-center pediatric SCD cohort, to characterize post-transplant complications, and to explore whether an age threshold for these complications can be identified.

## 2. Materials and Methods

We included, retrospectively, all patients under 20 years of age with SCD who underwent HCT in the Department of Pediatric Hematology and Oncology (Hôpitaux Universitaires de Strasbourg, France) between 2006 and 2024. No patient was excluded: the 33 patients correspond to all HCT performed in this population during the study period. All patients underwent HCT in Strasbourg, but four patients were referred from Reims, seven from Créteil, and one from Rouen and followed jointly by our department and the referring center. Data were collected from both electronic and paper medical records. This study was conducted in accordance with the Declaration of Helsinki. All patients and/or parents or guardians provided written informed consent.

Data are summarized as frequencies and percentages for categorical variables, and as medians and ranges for continuous variables. The probabilities of OS, EFS, GVHD-free rejection-free survival (GRFS), and survival free from arterial hypertension, posterior reversible encephalopathy syndrome (PRES), or veno-occlusive disease (VOD) were estimated using the Kaplan–Meier method. ROC curves were built using age at HCT as a continuous predictor; the cut-off maximizing the Youden index is reported with AUC, 95% confidence interval (CI), sensitivity and specificity, and number of events. Survival curves were compared with the log-rank test and median ages with the Mann–Whitney test.

For OS, death from any cause was retained. For TRM, only transplanted-related death was retained. Events retained for EFS were death from any cause, non-engraftment or rejection (donor cells < 5%). GRFS was defined as a composite endpoint of survival without grade III–IV acute GVHD, systemic therapy-requiring chronic GVHD, or recurrence of SCD symptoms. Neutrophil engraftment was defined as the first of two consecutive days with an absolute neutrophil count exceeding 0.5 × 10^9^/L. Platelet recovery was defined as the first of two consecutive days with an unsupported platelet count exceeding 50 × 10^9^/L. Patients were followed from HCT to last visit; patients without events were censored at last follow-up. VOD was diagnosed according to the criteria of the European Society for Blood and Marrow Transplantation (EBMT). Hypertension was defined as blood pressure ≥95th percentile for age, sex and height on at least three occasions and was retained as an outcome when antihypertensive treatment was required. Cerebral vasculopathy was defined as in the DREPAGREFFE protocol.

Donor chimerism was assessed by STR-PCR, on peripheral whole blood in MSD recipients and both on whole blood and the CD3+ fraction in haploidentical recipients at last follow-up (at least one year after HCT). Complete donor chimerism was defined as ≥ 95% donor cells.

Statistical analyses were performed using GraphPad Prism^®^ version 10.2.3 (GraphPad Software, San Diego, CA, USA). Means, standard deviations, and medians were calculated using Microsoft Excel.

## 3. Results

A total of 33 patients were included between 2006 and 2024; 30 received an MSD-HCT and 3 an haploidentical HCT.

### 3.1. MSD-HCT

#### 3.1.1. Patient Characteristics

Patient characteristics are summarized in Table 1.

The median age at transplantation was 8.4 years (3.7–16.7), and 47% of patients were female. SCD phenotypes included HbSS (90%) and HbSβ^0^ (10%). Most patients (70%) had received hydroxycarbamide prior to HCT, and almost half (43%) were on a chronic red blood cell transfusion program. Four patients had undergone splenectomy (13%). The primary indication for HCT was cerebral vasculopathy (43%), followed by acute chest syndrome (33%), recurrent VOC (10%), splenic sequestration (10%), and delayed hemolytic transfusion reaction (DHTR) (3%). No patient had pre-existing hypertension or a history of stroke before HCT.

#### 3.1.2. Donors and Cell Source

Most patients (73%) received bone marrow (BMT), three (10%) received cord blood (CBT), and five (17%) received the combination of both (CBT + BMT).

#### 3.1.3. Conditioning Regimen

All patients received intravenous busulfan from day −10 to day −6, with doses adjusted according to pharmacokinetic monitoring (AUC-based dosing), cyclophosphamide 50 mg/kg/day from day −5 to day −2 and thymoglobulin (rabbit antithymocyte globulin, ATG) 5 mg/kg/day from day −6 to day −3 (20mg/kg total dose).

#### 3.1.4. GVHD Prophylaxis

Patients received either cyclosporine A (CsA) alone or CsA combined with a short course of methotrexate (MTX) 10 mg/m^2^ on days +1, +3, and +6. CsA was initiated at 3 mg/kg/day and subsequently adjusted to target blood levels.

#### 3.1.5. Graft Characteristics and Engraftment

BMT allografts had a median total nucleated cell (TNC) count of 2.34 × 10^8^ cells/kg (0.88–8.00) and a median CD34^+^ cell count of 3.88 × 10^6^ cells/kg (0.56–8.64). Cord blood allografts had a median TNC count of 1.32 × 10^7^ cells/kg (0.7–4.6) and a median CD34^+^ cell count of 0.19 × 10^6^ cells/kg (0.003–2.72). The median time to neutrophil recovery after BMT, CBT + BMT and CBT was 21 days (13–52), 21 days (21–30) and 29 days (19–32), respectively. The median time from transplantation to the last platelet transfusions after BMT, CBT + BMT and CBT was 29 days (12–140), 24 days (21–37) and 54 days (30–115), respectively.

Among the patients with chimerism data available at least one year after transplantation (*n* = 29), 70% achieved complete donor chimerism (≥95% donor cells) and 30% had mixed chimerism (lowest value: 65%). No primary or secondary graft failure occurred in this cohort, and none of them showed SCD-related symptoms during follow-up, even those with a mixed chimerism.

#### 3.1.6. Outcomes

Five-year OS and EFS were both 93%, with one death among 30 patients (Figure 1A). The cause of death was accidental; therefore, TRM in this cohort was 0%. Median follow-up time was 4.1 years (0.8–16.4).

#### 3.1.7. GVHD

Two patients (7%) developed acute GVHD of grade II or higher: one presented with grade II cutaneous GVHD and one with grade IV cutaneous and digestive GVHD. One patient (3%) developed chronic GVHD with ocular involvement (dry eye syndrome, still ongoing). GRFS was 87% (Figure 1B).

#### 3.1.8. Infectious Complications

Eleven patients (37%) experienced a documented bacterial infection requiring hospitalization and intravenous antibiotics during or after hospitalization: 9 (82%) had sepsis, one (9%) had pyelonephritis.

EBV reactivation occurred in 22 patients (73%): 20 (91%) had PCR-positive reactivation without symptoms, and two (9%) developed EBV-associated lymphoproliferative disease. Eight (36%) required treatment with rituximab.

CMV reactivation (PCR-positive without symptoms) occurred in 16 patients (53%). Fourteen (88%) were treated, including seven by valganciclovir (44%); two by ganciclovir (13%); three by sequential ganciclovir and valganciclovir (19%); one by foscarnet (6%); and one by sequential foscarnet, ganciclovir, and valganciclovir (6%).

Six patients (20%) developed a fungal infection requiring treatment: two had fungemia (Candida albicans or Candida lusitaniae), two had oropharyngeal candidiasis, one had digestive candidiasis, and one had vaginal candidiasis. One patient developed intestinal giardiasis.

#### 3.1.9. Non-Infectious Complications

Post-transplant hypertension requiring treatment was observed in 53% of patients. Four patients (13%) developed PRES, confirmed by brain MRI; three of these four had no pre-existing cerebral vasculopathy. Two (7%) developed VOD: one severe and one mild.

#### 3.1.10. Age Threshold Analysis

ROC curve analyses were performed to identify an age threshold beyond which post-transplant complications would be more likely. For hypertension, the threshold was 9 years (events 16/30, AUC 0.53, *p* = 0.78, 95% IC 0.28–0.70, sensitivity 44%, specificity 50%), with a non-significant trend toward higher rates in older patients (*p* = 0.62, Figure 2A). For PRES (Figure 2B), the threshold was 8 years (events 4/30, AUC 0.62, *p* = 0.46, 95% IC 0.29–0.92, sensitivity 100%, specificity 50%), with a non-significant trend toward higher rates in older patients (*p* = 0.066, Figure 2B). For VOD, the threshold was 9 years (events 2/30, AUC 0.75, *p* = 0.24, 95% IC 0.37–1.00, sensitivity 100%, specificity 50%), with a non-significant trend toward higher risk below this threshold (*p* = 0.17, Figure 2C). Median age at transplantation did not differ significantly between patients with and without each complication: 8.4 vs. 8.6 years for hypertension (*p* = 0.97), 10.2 vs. 8.0 years for PRES (*p* = 0.46), and 6.5 vs. 9.0 years for VOD (*p* = 0.24; Mann–Whitney test).

Complication rates did not differ significantly between transplant periods (2006–2015 vs. 2016–2024).

#### 3.1.11. Cerebral Vasculopathy Outcomes

Pre- and post-transplant transcranial Doppler ultrasound or cerebral MRI angiography were performed in 15 patients. Post-transplant imaging was obtained at least one year after HCT, with the most recent study performed at last follow-up. Among the 13 patients transplanted for cerebral vasculopathy, 10 had an available imaging post-HCT: one showed radiological improvement on MRI, while the remainder were stable. In patients without prior vasculopathy, both MRI and transcranial Doppler findings remained normal. No worsening or new onset of post-transplant vasculopathy was observed. Among patients who developed PRES: the patient with pre-existing vasculopathy showed sequelae of PRES lesions alongside stable vasculopathy findings; two had normal MRI angiography, and one had pre-existing ischemic lesions related to SCD.

#### 3.1.12. Fertility

Endocrine follow-up data were available for 9 of the 14 females; the remaining patients were too young for pubertal assessment. Among these nine patients, eight had gonadal insufficiency and received hormone replacement therapy (three with estradiol gel, four with estradiol/dydrogesterone, and one with an estrogen patch). No fertility data were available for the 16 male patients.

### 3.2. Haploidentical HCT

Three patients received a haploidentical graft. Patients 1 and 2 were 16- and 19-year-old female patients, and Patient 3 was a 12-year-old boy. All had an SS phenotype, were on a transfusion program and received hydroxycarbamide prior to HCT; none of them had a splenectomy. All were transplanted for cerebral vasculopathy. All received thiotepa 10 mg/kg on day −7, fludarabine 30 mg/m^2^/day from day −6 to day −2, cyclophosphamide 14.5 mg/kg/day on days −6 and −5, ATG 0.5 mg/kg/day on day −9 and 2 mg/kg/day on days −8 and −7, and 2 Gy total body irradiation (TBI) on day −1, following the DREP-HAPLO protocol [7]; P3 was transplanted after the trial had closed to enrollment, while the other two were included in the trial. GVHD prophylaxis consisted of PT-Cy 50mg/kg on D + 3 and D + 4, sirolimus, and mycophenolate mofetil. All received BM grafts that had a median total TNC count of 2.46 × 10^8^ cells/kg (2.27–4.1) and a median CD34^+^ cell count of 2.09 × 10^6^ cells/kg (1.54–3.19).

P1 and P2 engrafted at D + 18 and D + 25, respectively, and achieved transfusion independence at D + 25 and D + 105, respectively. Both achieved 100% donor chimerism. P3 showed no engraftment, despite a CD34^+^-selected stem cell boost administered on D + 42 (CD34^+^ cell dose: 9.57 × 10^6^ cells/kg). This patient experienced primary graft failure, with 60% donor chimerism at D + 22 followed by complete rejection by D + 30 and subsequently underwent autologous reconstitution.

P1 developed grade II acute digestive GVHD, which resolved, but no cGVHD and experienced adenovirus, CMV, and EBV reactivation, as well as two episodes of pneumococcal meningitis. P2 had no GVHD and only experienced CMV reactivation. P3 experienced only an oral fungal infection and hypertension. None of them experienced VOD or PRES. Cerebral vasculopathy remained stable for all patients at 1-year after HCT, and neither female patient is currently receiving hormone replacement therapy.

## 4. Discussion

Regarding MSD-HCT patients, the median age at HCT was 8.4 years, consistent with previously published data. The main indication for HCT was cerebral vasculopathy. Most patients received BM graft following myeloablative conditioning with busulfan, cyclophosphamide and ATG with CsA and short-course methotrexate as GVHD prophylaxis. This study reports one accidental death (OS: 93%), no TRM and no rejection, in line with the national study by Bernaudin et al. [2] and other single-center European series [12,13]. These outcomes reinforce the recommendation of myeloablative allogeneic HCT for children with SCD who have an MSD.

In this exploratory analysis, we were unable to identify a statistically significant age threshold for hypertension, PRES, or VOD. With only 16, 4 and 2 events, respectively, the absence of statistical significance most likely reflects insufficient power rather than a true absence of effect. PRES tended to be more frequent in patients older than 8 years (*p* = 0.066), whereas VOD tended to be more frequent in younger patients (*p* = 0.17). Larger studies have shown that GVHD and poorer outcomes become more frequent after 13–15 years [2,11], and early transplantation has been advocated to avoid cumulative organ damage [14]. Our data are compatible with these recommendations, but documenting this in a larger cohort is warranted.

High rates of EBV reactivation (73%, with 36% requiring treatment) and CMV reactivation (53%, with 88% requiring treatment) were observed. CMV reactivation rates are comparable to those reported by Vallée et al. in a similar pediatric SCD cohort [15] and substantially higher than those documented in pediatric ALL transplant recipients (EBV 21.5%, CMV 32.3%) [16]. These elevated rates likely reflect ATG-induced immunosuppression, intentional tolerance of mixed chimerism in SCD resulting in delayed immune reconstitution and pre-existing functional asplenia. With the advent of letermovir prophylaxis in at-risk pediatric populations, CMV reactivation rates may decrease in future cohorts.

The incidence of GVHD was low, with a GRFS of 87%. Only one patient developed grade IV acute GVHD, and one patient retained active chronic ocular GVHD (dry eye syndrome). This favorable profile is likely attributable to the use of BM from MSD, which is well established as conferring lower GVHD risk [17].

Regarding cerebral vasculopathy outcomes, one patient demonstrated radiological improvement on MRI at least three years post-HCT, while it remainded stable in the others; further improvements may emerge with longer follow-up. These results are consistent with studies from St. Jude Children’s Hospital and Columbia University Medical Center, which reported stability of vascular lesions following pediatric allogeneic HCT [18,19]. The prospective DREPAGREFFE study further demonstrated that HCT improves cerebral vasculopathy over a three-year follow-up, compared with chronic transfusion therapy, which at best stabilizes the condition [20], and DREPAGREFFE-2 highlighted a significant decrease in stenosis and silent cerebral infarcts scores, as well as better working memory and processing speed, at 10 years post-transplant in the MSD-HCT arm compared with standard of care [21]. Our findings add to the literature supporting the use of HCT to halt vasculopathy progression and prevent silent cerebral infarction.

Impaired gonadal function is a recognized long-term complication of myeloablative conditioning. In our cohort, 8/9 female patients old enough to be assessed (out of 14 females) are receiving hormone replacement therapy for gonadal insufficiency. SCD itself contributes to hypofertility independently of conditioning through hypogonadism, abnormal sperm parameters in up to 91% of hydroxyurea-treated males [22], and reduced pregnancy rates in females [23]. Pre-transplant fertility preservation has become routine practice, though no validated technique for restoring spermatogenesis from cryopreserved testicular tissue has been established in humans, and clinical translation of spermatogonial stem cell transplantation remains investigational [24,25]. Whether RIC better preserves gonadal function remains uncertain: Rotz et al. found no significant difference in gonadal outcomes between MAC and RIC recipients [26], and prospective data from the DREPA-RIC study (closed 2024) are awaited.

For patients lacking an MSD, haploidentical HCT represents a potential alternative, although graft rejection remains a concern. Our small cohort cannot inform the general efficacy or safety of haploidentical HCT in children. Nevertheless, pediatric haploidentical HCT for SCD remains rare, and the primary graft failure occurring in our cohort documents an additional rejection outside the DREP-HAPLO trial in which a single rejection was observed. In our cohort, the 12-year-old patient who experienced primary graft failure had received a thiotepa-based conditioning regimen and PT-Cy following the DREP-HAPLO protocol. Notably, this patient was younger than the other two haploidentical recipients (12 vs. 16 and 19 years), consistent with findings from an international collaborative consortium, in which all graft rejections occurred exclusively in patients under 18 years of age, prompting a protocol amendment in the pediatric stratum [6].

Reported rejection rates in other haploidentical series vary according to conditioning regimen, ranging from 8 to 12.5% with non-thiotepa-based regimens [27,28] to 7% and 4.5% in thiotepa containing regimens [7,29], yet the graft rejection observed in our youngest haploidentical recipient (despite thiotepa and PT-Cy inclusion) raises the question of whether current conditioning provides sufficient immunosuppression in younger patients, in whom residual host immune reconstitution may be more robust, potentially supporting escalating thymoglobulin dosing in this population. This is a hypothesis for prospective evaluation, considering the infection risk and delayed immune reconstitution linked to higher ATG exposure. In parallel, TCRαβ/CD19-depleted haploidentical HCT has emerged as an appealing alternative, combining a low GVHD risk with rapid immune reconstitution mediated by γδ T cells and NK cells. In Corbacioglu’s team series, no graft rejection and no grade III–IV GVHD were observed in 20 SCD patients with a median age of 15 years [8,9]. However, an unacceptable 14% TRM related to infections was reported in this cohort, and prospective data from the ongoing multicenter trial (NCT04201210) are awaited to clarify whether this approach truly represents a suitable option for these patients.

This study is limited by its retrospective single-center design, temporal heterogeneity, small sample size, and resulting low statistical power. Incomplete follow-up data (particularly for male fertility) further constrain the conclusions on fertility in this population. We also noted that some patients missed follow-up appointments after transitioning to adult care, likely reflecting improved well-being post-HCT; this underscores the importance of structured transition programs and long-term prospective follow-up.

Lastly, emerging gene therapy approaches represent promising curative alternatives for patients without a suitable donor, though long-term efficacy and safety data are still accruing.

## 5. Conclusions

In this single-center retrospective pediatric cohort, MSD-HCT for SCD was associated with excellent OS and EFS with no TRM and low GVHD rates, consistent with national and European data. Our analysis did not identify a statistically significant age threshold for post-HCT complications; a possible trend around 8–9 years of age for PRES requires confirmation in larger studies. These results add to the established recommendation to perform HCT early in life, before the onset of irreversible disease-related complications. Regarding fertility, prospective studies including RIC-based protocols are needed to better characterize long term outcomes. The growing role of haploidentical transplantation warrants evaluation in larger prospective pediatric cohorts, with particular attention paid to conditioning strategies in children at higher risk of graft failure.

## Figures and Tables

**Figure 1:**
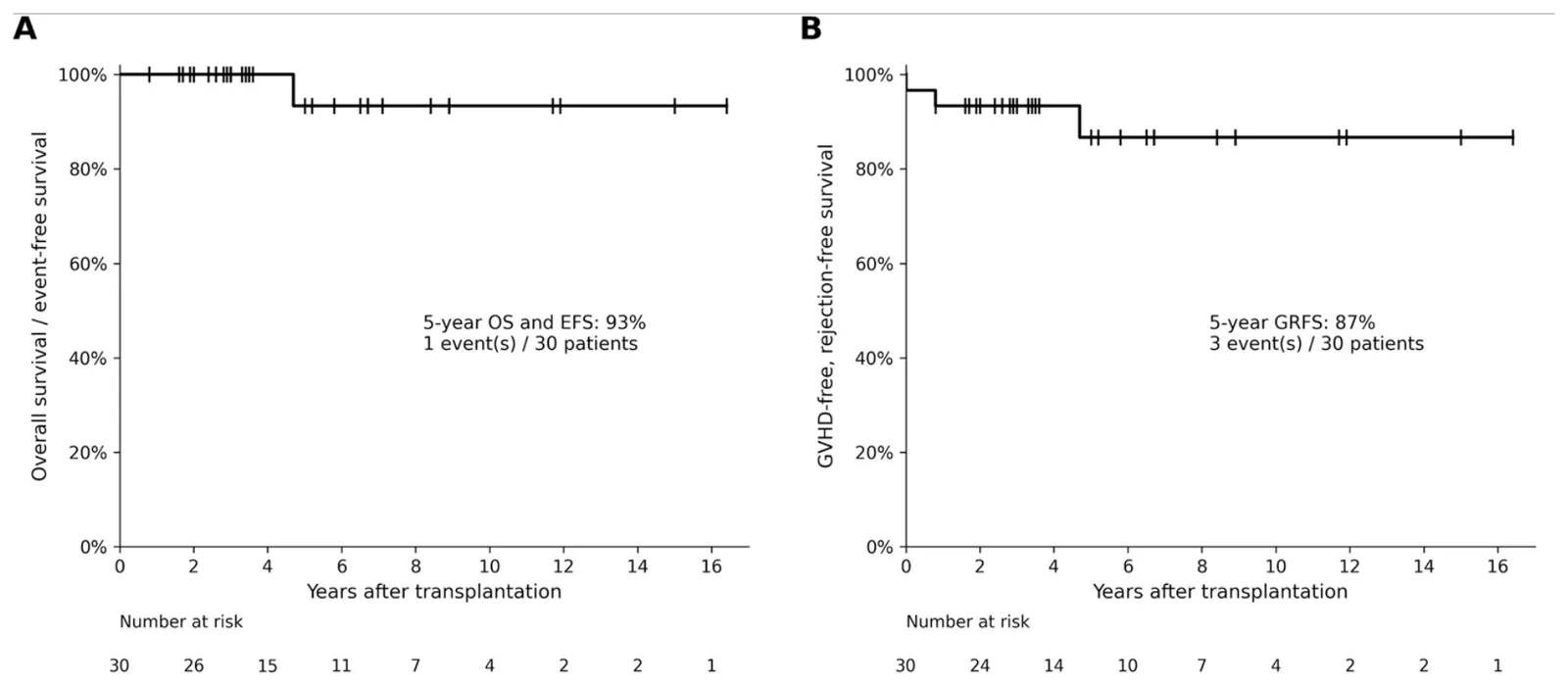
Overall and event-free survival (**A**) and GVH-free, rejection-free survival (**B**) of MSD-HCT patients.

**Figure 2:**
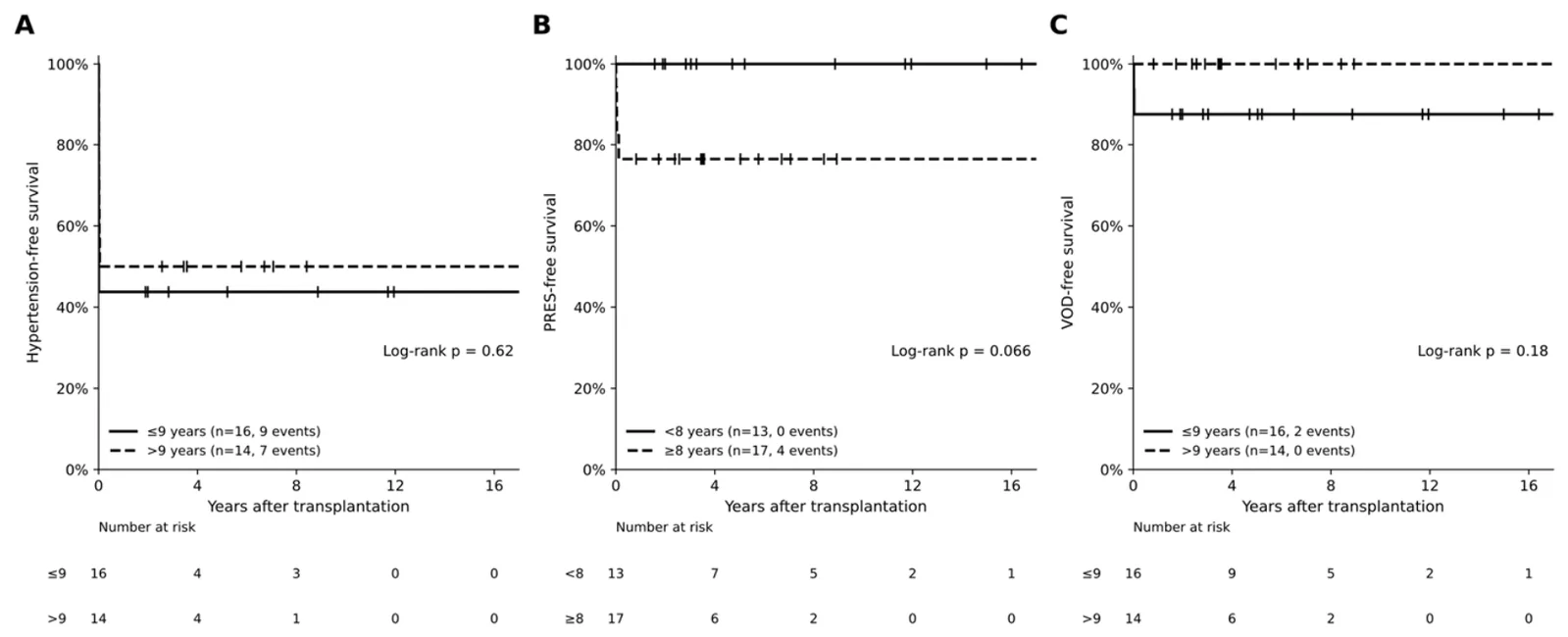
Kaplan–Meier estimates of survival free from (**A**) arterial hypertension, (**B**) posterior reversible encephalopathy syndrome (PRES) and (**C**) veno-occlusive disease (VOD) after MSD-HCT, according to age at transplantation. Tick marks indicate censored patients. The number of patients at risk is shown below each panel. P values are from the log-rank test.

**Table 1:** Characteristics of MSD-HCT recipients.

**Recipient Characteristics**	
Age, years, median (range)	8.4 (3.7–16.7)
<5 years old, *n* (%)	1 (3%)
5–13 years old, *n* (%)	24 (80%)
≥13 years old, *n* (%)	5 (17%)
Sex, *n* (%)	
Male	16 (53%)
Female	14 (47%)
Genotype	
SS	28 (93%)
Sß0	2 (7%)
Treatment with hydroxycarbamide prior to HCT, *n* (%)	21 (70%)
Transfusion program prior to HCT, *n* (%)	13 (43%)
History of ACS (at least one episode), *n* (%)	14 (47%)
**HCT characteristics**	
Indication, *n* (%)	
Repeated VOC	8 (10%)
VOC and ACS	10 (33%)
VOC and splenic sequestration	3 (10%)
Cerebral vasculopathy	13 (43%)
VOC and DHTR	1 (3%)
Stem cell source, *n* (%)	
Bone marrow	22 (73%)
Cord blood	3 (10%)
Bone marrow + cord blood	5 (17%)
GvHD prophylaxis, *n* (%)	
Cyclosporine alone	10 (33%)
Cyclosporine + methotrexate	20 (67%)
ATG	30 (100%)
**Donor characteristics**	
Age, years, median (range)	8.8 (0.6–20.4)

Abbreviations: ACS, acute chest syndrome; ATG, rabbit anti-thymocyte globulin; DHTR, delayed hemolytic transfusion reaction; GvHD, graft-versus-host disease; HCT, hematopoietic cell transplantation; MSD, matched sibling donor; and VOC, vaso-occlusive crisis.

## Data Availability

The raw data supporting the conclusions of this article will be made available by the authors on request.

## Abbreviations

The following abbreviations are used in this manuscript:

ALL	acute lymphoblastic leukemia
ATG	antithymocyte globulin
AUC	area under the curve (also used for busulfan exposure in AUC-based dosing)
BM	bone marrow
BMT	bone marrow transplantation
CBT	cord blood transplantation
CBT + BMT	combination of cord blood and bone marrow transplantation
cGVHD	chronic graft-versus-host disease
CMV	cytomegalovirus
CsA	cyclosporine A
DHTR	delayed hemolytic transfusion reaction
EBMT	European Society for Blood and Marrow Transplantation
EBV	Epstein–Barr virus
EFS	event-free survival
GRFS	GVHD-free, rejection-free survival
GVHD	graft-versus-host disease
HbSS	sickle cell hemoglobin, homozygous
HbSβ^0^	hemoglobin S/beta-zero thalassemia
HCT	hematopoietic cell transplantation
HLA	human leukocyte antigen
MAC	myeloablative conditioning
MRI	magnetic resonance imaging
MSD	matched sibling donor
MTX	methotrexate
NK	natural killer
OS	overall survival
P1, P2, P3	patient 1, 2, 3 (haploidentical recipients)
PCR	polymerase chain reaction
PRES	posterior reversible encephalopathy syndrome
PT-Cy	post-transplant cyclophosphamide
RIC	reduced-intensity conditioning
ROC	receiver operating characteristic
SCD	sickle cell disease
TBI	total body irradiation
TCRαβ	T-cell receptor alpha/beta
TNC	total nucleated cells
TRM	transplant-related mortality
VOC	vaso-occlusive crisis
VOD	veno-occlusive disease
